# Gear Shifting of Quadriceps during Isometric Knee Extension Disclosed Using Ultrasonography

**DOI:** 10.1155/2018/6385315

**Published:** 2018-03-18

**Authors:** Shu Zhang, Weijian Huang, Yu Zeng, Wenxiu Shi, Xianfen Diao, Xiguang Wei, Shan Ling

**Affiliations:** ^1^School of Biomedical Engineering, Shenzhen University, Shenzhen, China; ^2^Department of Electrical and Electronic Engineering, Hong Kong University, Pok Fu Lam, Hong Kong; ^3^Department of Biomedical Engineering, Case Western Reserve University, Cleveland, OH, USA

## Abstract

Ultrasonography has been widely employed to estimate the morphological changes of muscle during contraction. To further investigate the motion pattern of quadriceps during isometric knee extensions, we studied the relative motion pattern between femur and quadriceps under ultrasonography. An interesting observation is that although the force of isometric knee extension can be controlled to change almost linearly, femur in the simultaneously captured ultrasound video sequences has several different piecewise moving patterns. This phenomenon is like quadriceps having several forward gear ratios like a car starting from rest towards maximal voluntary contraction (MVC) and then returning to rest. Therefore, to verify this assumption, we captured several ultrasound video sequences of isometric knee extension and collected the torque/force signal simultaneously. Then we extract the shapes of femur from these ultrasound video sequences using video processing techniques and study the motion pattern both qualitatively and quantitatively. The phenomenon can be seen easier via a comparison between the torque signal and relative spatial distance between femur and quadriceps. Furthermore, we use cluster analysis techniques to study the process and the clustering results also provided preliminary support to the conclusion that, during both ramp increasing and decreasing phases, quadriceps contraction may have several forward gear ratios relative to femur.

## 1. Introduction

Muscle behavior in vivo is an essential problem to be resolved. Commonly used techniques for measuring muscle activities from different aspects include electromyography (EMG) [1], dynamometers, and ultrasonography [2]. Surface EMG is the most widely used tool for indirect assessment of mechanical activity of muscle, but it fails to disclose muscle's morphological changes. Dynamometers are devices for quantitative measurement of muscle, such as torque and power, but they cannot provide muscle's morphological information yet. Due to the quality of real-time imaging, widespread availability, and low cost, ultrasonography has been increasingly employed as a clinical and research tool to study the in vivo behavior of the quadriceps muscle from the morphological point of view, such as architectural changes of thickness [3–6], fascicle length [2, 6–10], pennation angle [2, 7, 8, 10–12], and cross-sectional area [2, 13, 14]. Dick and Wakeling recorded medial gastrocnemius tendon length, fascicle length, pennation angle, and thickness using ultrasonography and muscle activation using surface EMG during cycling [15]. They identified muscle force, and not velocity, as the mechanistic driving factor to allow muscle gearing to vary depending on the contractile conditions. All these works show the potential of using data and quantitative approaches to help understand the nature and functional implications of in vivo dynamic body movement. Quadriceps is always a focus as it is crucial in walking, running, jumping, and squatting. Wei et al. reported an image-based method to find the contour of the center tendon of rectus femoris quantitatively [16]. However, the knowledge about relative movement between quadriceps and femur during dynamic contraction remains unclear. The aim of this study is to quantify this movement for further understanding of muscle contraction mechanism.

As a matter of fact, in the data from a previous study [16], a phenomenon like shifting gear of a car was observed visually from time to time. Simply speaking, when the torque increases linearly, femur is frequently noticed to move around several relatively fixed positions. To verify this assumption, we repeated the experiment and recorded both the torque signal and the movement of femur under ultrasonography. Then, some established image processing techniques were used to disclose the gear shifting phenomenon.

## 2. Materials and Methods

### 2.1. Subjects and Experiment Protocol

Eight healthy male subjects (mean ± SD, age = 28.5 ± 0.6 years; body weight 67.3 ± 1.7 kg; height = 171.8 ± 0.6 cm) volunteered to participate in this study. No participants had a history of neuromuscular disorders, and all were aware of experimental purposes and procedures. The human subject ethical approval was obtained from the relevant committee in the authors' institution, and informed consent was obtained from subjects prior to the experiment.

The testing position of the subject was in accordance with the User's Guide of a Norm dynamometer (Humac/Norm Testing and Rehabilitation System, Computer Sports Medicine, Inc., Massachusetts, USA). Each subject was required to put forth his maximal effort of isometric plantar flexion for a period of 3 seconds with verbal encouragement provided. The maximal voluntary contraction (MVC) was defined as the highest value of torque recorded during the entire isometric contraction. The MVC torque was then calculated by averaging the two recorded highest torque values from the two tests. The subject was instructed to generate a torque waveform up to 90% of his MVC, using ankle plantar flexion movements in prone position. The torque was measured by the aforementioned dynamometer and the reason for choosing 90% MVC as the highest value was to avoid muscle fatigue.

### 2.2. Data Acquisition and Data Processing

**Figure 1:**
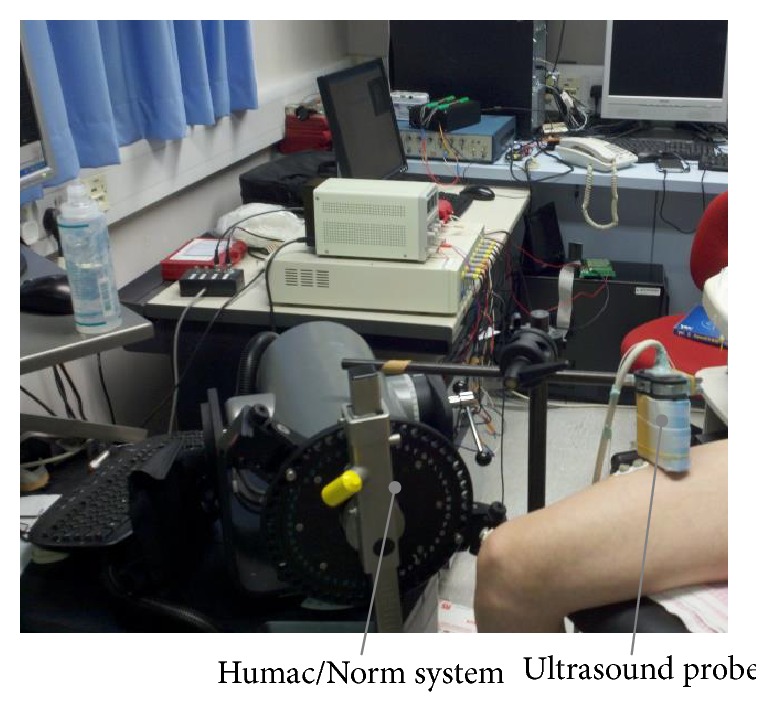
Experimental setup including the torque and ultrasound image data collection modules.

A real-time B-mode ultrasonic scanner (EUB-8500, Hitachi Medical Corporation, Tokyo, Japan) with a 10 MHz electronic linear array probe (L53L, Hitachi Medical Corporation, Tokyo, Japan) was used to obtain ultrasound images of muscles. The long axis of the ultrasound probe (EUB-8500) was arranged perpendicularly to the long axis of the thigh on its superior aspect, 40% distally from the knee. As the position of probe-quadriceps is fixed, the movement of femur reflects the contraction of quadriceps. The ultrasound probe was fixed by a custom-designed foam container with fixing straps, and a very generous amount of ultrasound gel was applied to secure acoustic coupling between the probe and skin during muscle contractions, as shown in Figure 1. The probe was adjusted to optimize the contrast of muscle fascicles in ultrasound images. Then the B-mode ultrasound images were digitized by a video card (NI PCI-1411, National Instruments, Austin, USA) at a rate of 25 frame/s for later analysis.

Eight sequences of musculoskeletal ultrasound images were acquired and the number of frames per sequence was 240 images. All images were cropped to remove equipment tags in the images and keep the image content only using a home-made software. All data were processed offline using programs written in Matlab R2010b (Math Works, Natick, MA, USA) on a Windows-based computer with a P4 (3 GHz) processor and 2 GB memory.

### 2.3. Image Filtering and Femur Segmentation

In this study, the shapes of femur are extracted automatically by two steps. The ultrasound images are first denoised using guided filter. Then, the femur in a sequence is segmented by using an active contour model, named implicit active contours driven by local binary fitting energy (LBF) [17]. To reduce the computation time and improve the accuracy of femur extraction, we located the region of interest (ROI) as a rectangle which could enclose the femur of the whole image sequence, and the subsequent operations are applied on this region rather than the whole image. The rectangle is expected to be small but able to contain the femur of the sequence.

Ultrasound images are usually affected by speckle noise [18], and the edges of femur are not clear in most images, which make it hard for the segmentation algorithm to recognize the accurate boundaries of femur. To handle this problem, we use a filtering algorithm, named guided filtering, for image smoothing and noise reduction before the segmentation step. This filter has edge-preserving smoothing property like bilateral filter but does not suffer from the gradient reversal artifacts. The derivation and details of the guided filtering algorithm can be found in Appendix A.

Although images are smoothed after guided filtering, intensity inhomogeneity still exists in ultrasound frames. Hence, we adopted implicit active contours driven by local binary fitting energy (LBF), to extract boundaries of femur from ultrasound images. In this model, a kernel function is introduced into a data fitting energy, so that intensity information in local regions is extracted to guide the motion of the contour, which thereby enables the model to cope with intensity inhomogeneity. The details and implementation of the LBF model are introduced in Appendix A. In this study, level set of each frame is initialized as two rectangles enclosing the femur. Then contours of femur will be extracted by evolving the level set iteratively using LBF model.

### 2.4. Analysis of Femur Motion

To study the motion of femur, we use the contours in the previous step to generate corresponding segmentation images for the femur.  Figure 5 is an overlapped plot of segmentation results in a knee extension process. In this figure, different color represents the duration time of the femur staying at this location and one may easily observe that the femur mainly stays at several positions.  Figure 6 is a schematic drawing of a cross-sectional view of the quadriceps in which A, B, and C are different stages of femur motion. To see the motion in detail, we draw the overlapped plot of femur movement in both muscle contraction and relaxation stages in Figure 8(a) and plot an example of torque signal and related *x*  *y* coordinates in Figures 8(b) and 8(c). In order to further study the process, we used optimal detection of change-points (ODC) algorithm [19] to cut the process into different clusters. The reason of using ODC is that the motion is a temporal continuous process and ODC algorithm can cover this continuity by design. The results and discussion of clustering can be found in the next section.

**Figure 2:**
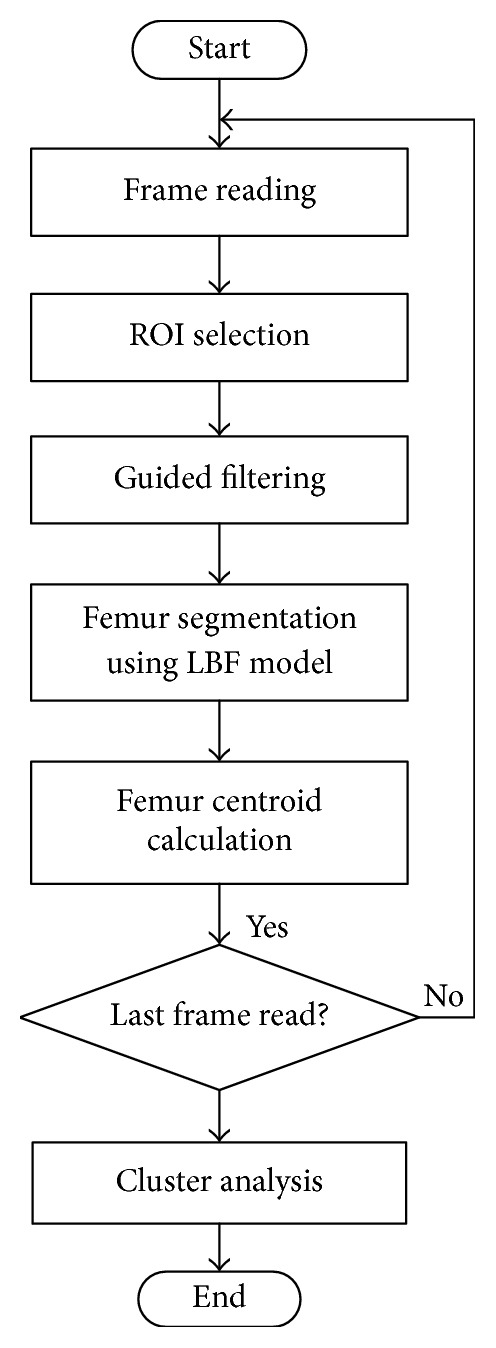
Flowchart of the proposed strategy for processing an ultrasound image sequence.

The flowchart of the proposed strategy mentioned above is illustrated in Figure 2.

## 3. Results and Discussion

### 3.1. Image Filtering and Femur Segmentation

After acquisition of ultrasound frames, images were focused on the ROI, and then guided filter and LBF model were applied to smooth the image and extract the femur, respectively. In our numerical experiments, window radius *r* and regularization parameter *ε* for guided filter are selected as 8 and 0.4^2^, respectively. And for femur segmentation using LBF model we empirically adopted parameters as follows: *λ*_1_ = 1, *λ*_2_ = 2, *ν* = 0.001 × 255 × 225, *μ* = 1, *σ* = 10, and time step Δ*t* = 0.1. All parameters are empirical values and applied to frames of all subjects. The explanation and usage for mentioned parameters can be found in the Appendices.

**Figure 3:**
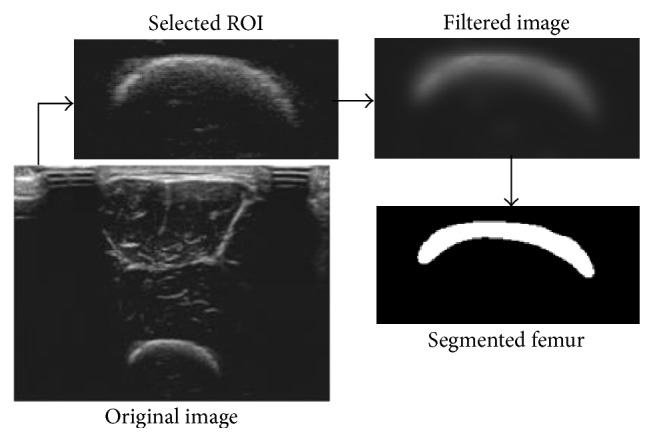
A representative result of ROI selection, guided filtering, and femur segmentation.

**Figure 4:**
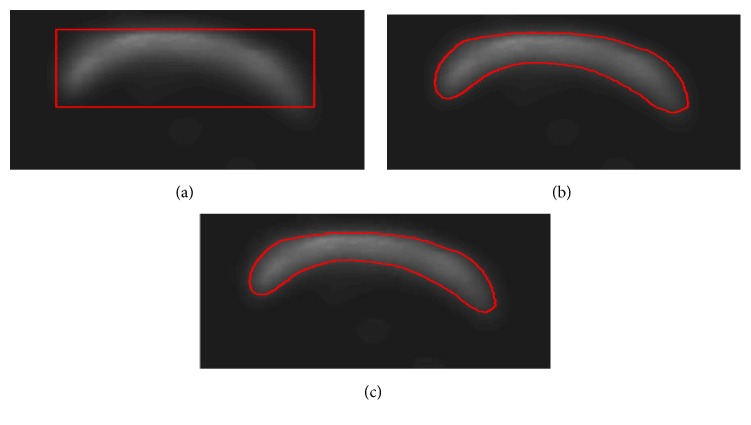
A representative set of results of LBF model for femur segmentation. (a) Original image with initial contour. (b) Curve evolution result after 2 iterations. (c) Final contour after evolving stopped.

A representative example of selected ROI, filtered image, and segmented femur are shown in Figure 3. And a representative example of femur segmentation process using LBF evolution model is shown in Figure 4.

### 3.2. Discussion

**Figure 5:**
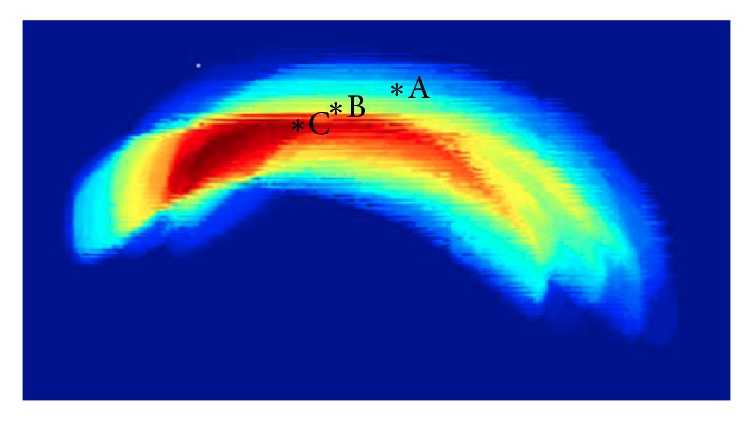
A representative statistical result of femur position in a sequence, where *∗*A,  *∗*B, and *∗*C are the positions the femur mainly stay at.

**Figure 6:**
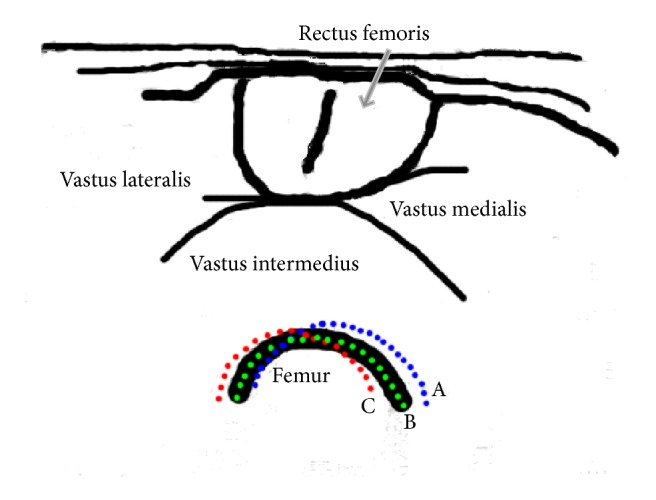
Schematic drawing of a cross-sectional view of the quadriceps shows the femur movement during a contraction-relaxation process of quadriceps, where A, B, and C are the main stages of femur movement.

After segmentation of femur, an interesting phenomenon could be found where (shown in Figure 5), corresponding to one ramp increasing and decreasing of quadriceps, femur relative to the ultrasound sensor in a sequence mainly concentrated at several positions which are shown as *∗*A, *∗*B, and *∗*C. To further investigate the detailed motion and its relationship to torque changing, in Figure 8, we draw the overlapped plot of femur movement in both muscle contraction and relaxation stages in (a), plot the torque signal in (b), and plot the *x*  *y* coordinates of the femur centroid movement in (c). Then we use optimal detection of change-points (ODC) method [19] to cut whole process into different stages using torque signal and *x*  *y* coordinates. We can see the motion of femur and the torque changing have very different behaviors. The torque signal, which is shown in Figure 8(b), can be mainly divided into three stages which are corresponding to muscle contraction, relaxation, and resting, respectively, and, in each stage, the torque changes almost linearly, while, in Figures 8(c) and 8(d), the plotted *x* and *y* coordinates of femur centroid movement can be viewed as more stages than torque changing. When the torque signal changes into another stage, the motion of femur also changes to another stage which makes sense. Interestingly, we can observe that when the torque linearly increases or decreases in a stage, the trend of femur movement is not fixed and can be further divided into several stages. This stage changing reminds us of gear shifting in a car where there might also be a gear shifting of femur in the extension process. Therefore, to further investigate this phenomenon quantitatively, we use cluster analysis methods to study the behavior of femur movement. We first cut the process using torque signal into three stages, that is, contraction, relaxation, and resting. (The contraction stage and relaxation stage are separated by peak torque while the relaxation stage changes to resting stage when the torque signal reduced to less than 0.05.)

**Figure 7:**
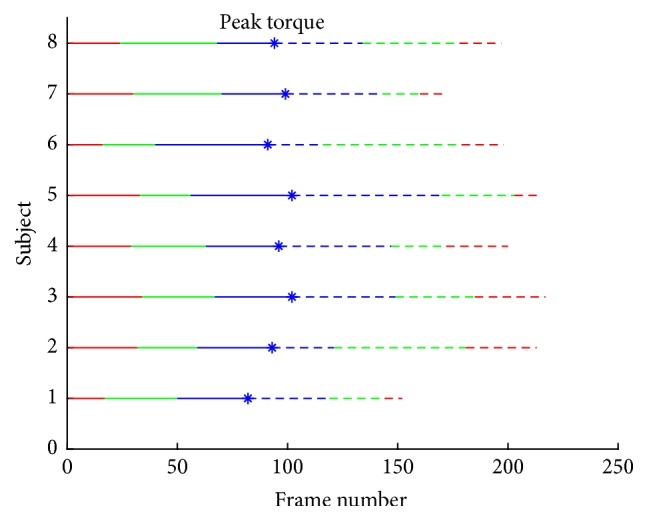
Classification results of all subjects.

**Figure 8:**
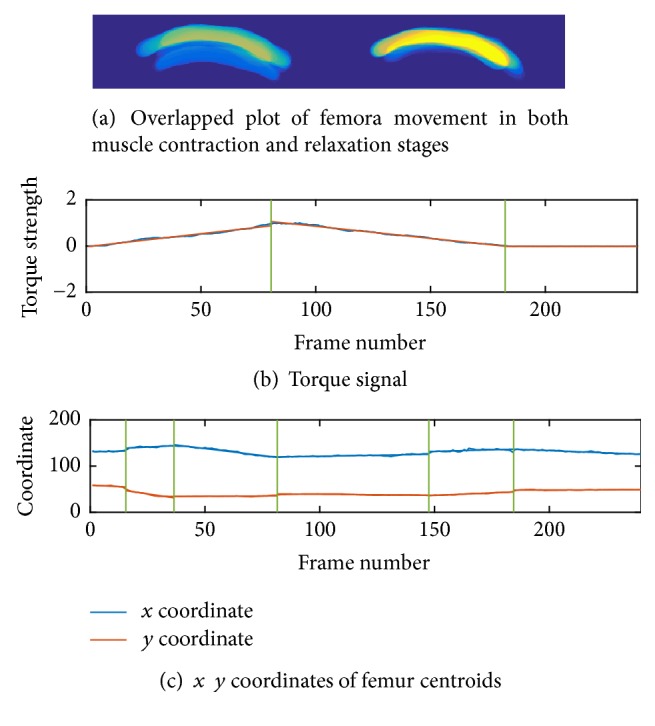
(a) Overlapped plot of muscle contraction and relaxation stages, (b) torque signal, and (c) *x*  *y* coordinates of femur centroids.

As we are studying the extension process, we only focus on contraction and relaxation stages. Both contraction and relaxation stages are clustered into three phases by ODC. Clustering results are displayed in Figure 7 and the six clusters are represented by #A, #B, #C, #C′, #B′, and #A′, in which #A, #B, and #C correspond to beginning, middle, and ending of contraction stage and #A′, #B′, and #C′ correspond to beginning, middle, and ending of relaxing stage. In Table 1, we first summarized the variances of each cluster in all subjects. In this table, we also included their rankings. Interestingly, in the relaxation phase, we find that #B′ has the largest variances for 7/8 subjects, #C′ has the median variances for 6/8 subjects, and #A′ has the smallest variances for 7/8 subjects. The variance of a group represents the moving speed of the femur in this cluster and we may conclude that the moving speed of femur changes in a fixed pattern in relaxation phase. In the contraction phase, the property is not very clear, but we can still notice that the femur generally moves faster in #A than in #B and #C. At all events, from the clustering results, we can find that the clusters in the same contraction or relaxation phase have different properties whereas the torque signal keeps linear changing. #A and #A′, #B and #B′, and #C and #C′ are considered as pairs as they have similar torque signal and, therefore, in Table 2, we also summarized the distances among them. Another interesting phenomenon can be seen from Table 2 that distances between clusters #A and #A′ are much larger than distances of other pairs. This can support the early suggestion that complete relaxation of muscle takes time. In many cases, muscle morphology has not returned to the initial condition although torque has done.

To sum up the points which we have just indicated, the main finding of this study could conclude that quadriceps movement is nonlinear and the relative position between the quadriceps and femur is piecewise with the change of torque during one contraction-relaxation. In other words, the contraction of quadriceps may have gear shifting mechanism during isometric knee extension.

## 4. Conclusion

In this paper, we observed a gear shifting pattern of quadriceps. To validate our observation, we proposed a systematic strategy to analyze the isometric knee extension process via ultrasonography, video processing, and related signal processing techniques. Analysis results provide preliminary support for the phenomenon. To our knowledge, the present study is the first report to describe the gear shifting motion pattern during quadriceps contractions in human skeletal muscles.

However, there are still several limitations of this study. The number of participants is small. In future work, larger dataset would allow making further validation.

## Figures and Tables

**Table 1:** Variances of clusters.

Subject	#A		#B		#C		#C′		#B′		#A′	
1	15.4	Medium	18.7	Large	3.9	Small	8.6	Medium	24.0	Large	5.5	Small
2	3.9	Small	26.8	Medium	76.5	Large	2.3	Small	18.8	Large	8.6	Medium
3	19.0	Large	4.3	Small	15.3	Medium	5.8	Small	36.0	Large	10.1	Medium
4	28.1	Medium	6.7	Small	55.6	Large	7.1	Small	10.3	Medium	11.0	Large
5	27.0	Large	5.0	Medium	3.1	Small	8.5	Small	19.0	Large	14.2	Medium
6	69.9	Large	14.1	Medium	1.3	Small	3.4	Small	41.9	Large	10.2	Medium
7	7.2	Small	17.9	Large	15.7	Medium	4.0	Small	24.3	Large	9.7	Medium
8	16.2	Large	7.3	Small	15.1	Medium	4.0	Medium	31.7	Large	4.0	Small

**Table 2:** Cluster distance between contraction group and relaxation group.

Subject	Euclidean distance (mm)		
	#A-#A′	#B-#B′	#C-#C′
1	17.3	13.6	3.1
2	14.6	13.5	10.8
3	16.9	2.1	6.6
4	12.6	17.5	8.3
5	26.2	4.7	5.8
6	12.1	2.7	2.3
7	2.9	9.8	6.6
8	5.2	0.4	6.7
Mean	11.3	5.0	5.7

## Appendix

### A. Details of Guided Filtering Algorithm

The key assumption of the guided filter is that the filter output *q* is a linear transform of the guidance image *I* in a window *ω*_*k*_ centered at the pixel *k*:

(A.1) $$\begin{array}{c} q_{i}=a_{k}I_{i}+b_{k},\;\forall i\in \omega_{k}, \end{array}$$

where (*a*_*k*_, *b*_*k*_) are some linear coefficients assumed to be constant in a square window *ω*_*k*_ with a radius *r*.

To determine the linear coefficients, the following cost function is minimized in the window:

(A.2) $$\begin{array}{c} E\left(\;a_{k},b_{k}\;\right)=\underset{i\in \omega_{k}}{\sum }\left(\;{\left(\;a_{k}I_{i}+b_{k}-p_{i}\;\right)}^{2}+\varepsilon {a_{k}}^{2}\;\right). \end{array}$$

Here *ε* is a regularization parameter, *I* and *q* are identical and given as the input ultrasound image. The solution to (A.2) can be given by linear regression:

(A.3) ak=1/ω∑i∈ωkIipi−μkp−kσk2+εbk=p−k−akμk.

Here, *μ*_*k*_ and *σ*_*k*_^2^ are the mean and variance of *I* in *ω*_*k*_, |*ω*| is the number of pixels in *ω*_*k*_, and ${\overset{-}{p}}_{k}=\left(1/\left|\omega \right|\right)\sum_{i\in \omega_{k}}^{}p_{i}$ is the mean of *p* in *ω*_*k*_.

So after computing (*a*_*k*_, *b*_*k*_) for all patches *ω*_*k*_ in the image, we compute the filter output by

(A.4) $$\begin{array}{c} q_{i}=\frac{1}{\left|\omega \right|}\overset{}{\underset{k:i\in \omega_{k}}{\sum }}\left(\;a_{k}I_{i}+b_{k}\;\right). \end{array}$$

### B. Details of Local Binary Fitting Model

Consider the input image *I* : *Ω* → *𝔑*^2^, where *Ω* is the image domain. Let *ϕ* be the level set of a Lipschitz function; the gradient decent flow equation of the LBF energy functional is defined as

(B.1) ∂ϕ∂t=−δεϕλ1e1−λ2e2+νδεϕ·div⁡∇ϕ∇ϕ+μ∇2ϕ−div⁡∇ϕ∇ϕ,

where *λ*_1_, *λ*_2_, *ν*, and *μ* are positive constants, *K*_*σ*_ is a Gaussian kernel function with parameter *σ*, *δ*_*ε*_ is the smooth Dirac function with parameter *ε* = 1.0, and *e*_1_ and *e*_2_ are the functions as follows:

(B.2) e1x=∫ΩKσy−xIx−f1y2dye2x=∫ΩKσy−xIx−f2y2dy.

where *K*_*σ*_ is the Gaussian kernel with standard deviation *σ*, and *f*_1_, *f*_2_ are given by

(B.3) f1x=Kσx∗HεϕxIxKσx∗Hεϕxf2x=Kσx∗1−HεϕxIxKσx∗1−Hεϕx.

In practice, the Heaviside function *H* is approximated by a smooth function *H*_*ε*_ defined by

(B.4) $$\begin{array}{c} H_{\varepsilon }\left(\;x\;\right)=\frac{1}{2}\left[\;1+\frac{2}{\pi }\arctan\left(\;\frac{x}{\varepsilon }\;\right)\;\right]. \end{array}$$

The derivative of *H*_*ε*_ is the following smooth function:

(B.5) $$\begin{array}{c} \delta_{\varepsilon }\left(\;x\;\right)=H_{\varepsilon }^{\prime }\left(\;x\;\right)=\frac{1}{\pi }\frac{\varepsilon }{\varepsilon^{2}+\pi^{2}}. \end{array}$$
